# Non-Coding RNAs and Hereditary Hemorrhagic Telangiectasia

**DOI:** 10.3390/jcm9103333

**Published:** 2020-10-17

**Authors:** Anthony Cannavicci, Qiuwang Zhang, Michael J. B. Kutryk

**Affiliations:** 1 Institute of Medical Science, University of Toronto, Toronto, ON M5S 1A8, Canada; a.cannavicci@mail.utoronto.ca; 2 Division of Cardiology, Keenan Research Center for Biomedical Sciences, St. Michael’s Hospital, Unity Health Toronto, University of Toronto, Toronto, ON M5B 1T8, Canada; Qiuwang.Zhang@unityhealth.to

**Keywords:** non-coding RNAs, microRNAs, long non-coding RNAs, hereditary hemorrhagic telangiectasia, biomarkers, endothelial cells, angiogenesis

## Abstract

Non-coding RNAs (ncRNAs) are functional ribonucleic acid (RNA) species that include microRNAs (miRs), a class of short non-coding RNAs (∼21–25 nucleotides), and long non-coding RNAs (lncRNAs) consisting of more than 200 nucleotides. They regulate gene expression post-transcriptionally and are involved in a wide range of pathophysiological processes. Hereditary hemorrhagic telangiectasia (HHT) is a rare disorder inherited in an autosomal dominant fashion characterized by vascular dysplasia. Patients can develop life-threatening vascular malformations and experience severe hemorrhaging. Effective pharmacological therapies are limited. The study of ncRNAs in HHT is an emerging field with great promise. This review will explore the current literature on the involvement of ncRNAs in HHT as diagnostic and pathogenic factors.

## 1. Introduction

Hereditary hemorrhagic telangiectasia (HHT) is a rare genetic vascular disorder inherited in an autosomal dominant fashion. On average, approximately one in 5000 to 8000 people are affected, while the founder effect has contributed to a higher prevalence in certain regions, such as the Netherlands Antilles, Jura in France and Funen in Denmark [1]. Vascular malformations in HHT include skin and mucocutaneous telangiectasias, and pulmonary, cerebral, hepatic and spinal arteriovenous malformations (AVMs) [2,3], all of which are susceptible to rupture with resultant spontaneous hemorrhage. Epistaxis is the most common symptom and is present in approximately 95% of patients [4,5]. HHT is a progressive disorder with significant morbidities and mortality, and lacks a universally effective pharmacological therapy [2].

HHT is caused by heterozygous mutations in at least three known genes: endoglin (*ENG*, chromosomal locus 9q34) [6], activin receptor-like kinase 1 (*ACVRL1*, also known as *ALK1*, chromosomal locus 12q1) [7] and mothers against decapentaplegic homolog 4 (*SMAD4*, chromosomal locus 18q21) [8]. Each gene encodes for a protein in the transforming growth factor beta (TGFβ)/bone morphogenetic protein (BMP) signaling pathway. This pathway is responsible for many cellular functions, including growth, differentiation and apoptosis, and is critical in angiogenesis and normal endothelial cell (EC) function [9]. The pathogenic role of these genes has been demonstrated in the adult mouse where the homozygous knockout of *ENG*, *ACVRL1* or *SMAD4* resulted in various vascular defects, including AVMs [10,11]. *ENG* encodes for a TGFβ co-receptor that enhances the affinity of ligand binding to TGFβI and II receptors. This co-receptor is predominately expressed on the endothelium, activated monocytes and macrophages [2]. *ACVRL1* encodes for a TGFβ1 receptor that is predominately expressed on endothelial, lung and placental cells [2]. Mutations in *ENG* and *ACVRL1* result in HHT1 and HHT2, respectively, and on average display distinct clinical manifestations, but overlap is not uncommon. Sabbà et al. demonstrated a higher prevalence of pulmonary (75.5% vs. 44.1%) and cerebral AVMs (20.9% vs. 0%) in HHT1, while liver manifestations were higher in HHT2 (83.1% vs. 60%) [12]. SMAD4 is a signal transducer in the TGFβ signaling pathway that directly regulates gene expression. Mutations in *SMAD4* not only result in HHT, but juvenile polyposis (JP), culminating in a combined syndrome designated as JP/HHT [8]. *ENG* and *ACVRL1* mutations are responsible for 90% of HHT cases, while *SMAD4* contributes to only 2% [13,14]. A small percentage of cases have been attributed to novel disease loci, HHT 3 (chromosomal locus 5q31) [15] and HHT 4 (chromosomal locus 7p14) [16], but these genes have yet to be identified. Over 700 pathogenic mutations have been identified in *ENG* and *ACVRL1* patients (https://arup.utah.edu/database/HHT/, access date: 20/04/2020), comprising single base pair changes, large deletions, duplications, substitutions and missense mutations [13,17]. Interestingly, disease severity and the presentation of clinical manifestations vary drastically between patients and this is further demonstrated in affected family members. This discrepancy suggests that the genetic mutations alone are not entirely responsible for disease characteristics and raises the question: what other biological factors could be at play?

Non-coding RNAs (ncRNAs) are functional ribonucleic acid (RNA) sequences that are transcribed from DNA, but not translated into protein. NcRNAs can be divided into three categories based on their length: (1) ncRNAs longer than 200 nucleotides (nts), including ribosomal RNA (rRNA), long non-coding RNA (lncRNA) and circular RNA (circRNA); (2) ncRNAs shorter than 200 nts, but longer than 40 nts, such as transfer RNA (tRNA), small nucleolar RNA (snoRNA), Ro-associated Y RNA (YRNA) and small nuclear ribonucleic acid RNA (snRNA); and (3) ncRNA shorter than 40 nts like microRNA (miRNA), piwi-interacting RNA (piRNA), short interfering RNA (siRNA) and tRNA-derived small RNA (tsRNA) [18]. NcRNAs regulate gene expression at the transcriptional and post-transcriptional levels, and are involved in a wide array of cellular processes. In particular, snRNAs and snoRNAs are involved in mRNA maturation; rRNAs and tRNAs are important components for protein translation and miRNAs, piRNAs and lncRNAs are involved in the regulation of target gene expression.

MiRNAs (miRs) are the best studied group of noncoding RNAs. They were first described in 1993 by the Ambros and Ruvkun groups and have since caused a paradigm shift in how we understand biological processes [19,20]. Over 2000 miRs have been identified and it has been postulated that they regulate 30% of known genes [20,21]. Processed by endonucleases, the single-stranded miRs bind to cognate mRNAs to induce translational silencing by altering transcript stability or impacting mRNA translation. A single miR can have multiple mRNA targets. MiRs are involved in almost every cellular process [22] and play a role in a wide range of human diseases [22,23]. They have been proven to have reliable diagnostic and prognostic attributes [24], especially in oncology, and are being pursued as potential therapeutic targets [25,26]. A growing class of miRs known as “angio-miRs” have also been shown to contribute to vascular diseases [27]. Given that HHT is a disorder characterized by vascular dysfunction, it is possible that “angio-miRs” play a role in HHT pathogenesis. However, the exact role of any class of miR has yet to be fully characterized in HHT. In this review, we discuss our current understanding of the involvement of ncRNAs in HHT as circulating biomarkers, pathogenic factors and the potential for ncRNAs as therapeutic targets.

## 2. MiR Biogenesis and Mechanisms of Action

Nearly half of all identified miRs are expressed from specific genes with their own promoters, with the remainder from protein-coding genes [28,29]. Additionally, multiple miRs may be expressed as a single transcript, defined as families or clusters, and share similar target homology [30]. Canonical miR biogenesis is initiated with miR gene transcription by an RNA polymerase II [31]. The result is an ~80 nucleotide stem–loop structure called a primary miR (pri-miR) [32]. The pri-miR is further processed by an RNase III enzyme and a double-stranded RNA-binding protein (dsRBP), called Drosha and DGCR8, respectively [32]. This complex shortens the pri-miR to ~70 nts to generate the pre-miR. The pre-miR is exported from the nucleus to the cytoplasm where it is once again processed by an RNase III enzyme and dsRBP, Dicer and transactivation-responsive (TAR) RNA-binding protein (TRBP), respectively, effectively removing the hairpin to produce a miR–miR duplex [32,33]. The duplex comprises a passenger strand and a guide strand. The passenger strand is degraded, while the guide strand is incorporated into the RNA-induced silencing complex (RISC). Strand incorporation is dependent on the thermodynamic stability of the 5′ end of the miR–miR duplex, where the less stable strand is incorporated [34]. RISC contains an RNA binding protein responsible for miR silencing activity, Argonaute 2 (AGO2). AGO2 has potent RNase-H-like endonuclease activity and is capable of cleaving mRNAs [35].

MiRs guide the RISC–AGO complex to target mRNAs by recognizing the miR response element (MRE) in the 3′ untranslated region (UTR) [36,37]. Alterative binding sites have been identified in the 5′ UTR, coding sequences and within promoter regions [37]. Base pair complementarity between miRs and MREs dictates the mode of gene silencing. Perfect complementarity activates AGO2′s endonuclease activity, resulting in the cleavage and subsequent degradation of target mRNAs [35]. However, in metazoans, this rarely occurs and the majority of miR–MRE interactions are not perfectly complementary [38]. In this scenario, translational inhibition can occur where the miR–RISC–AGO complex likely blocks translational machinery from binding. Alternatively, proteins in complex with AGO2 can recruit poly(A)-deadenylases to elicit mRNA degradation [38]. In this way, miRs can regulate the expression of tens to hundreds of mRNAs. Bioinformatic analyses can use algorithms to predict complementarity for the identification of hundreds of mRNA targets per miR, but realistically only a small subset are experimentally validated [21]. It is important to note that individual miR activity is dependent on several factors, including miR tissue expression profiles (tissue-specific vs. housekeeping) and miR expression levels. Typically, higher miR expression will have a more robust effect on target mRNAs.

## 3. Circulating MiR Biomarkers in HHT 

The discovery of circulating miRs was achieved by several groups [39,40,41], most notably by Chim et al. who identified that stable plasma miRs could distinguish between pregnant and non-pregnant women [42]. Since then, circulating miRs have been established as stable and sensitive candidate biomarkers for various diseases, including cancers, cardiovascular diseases and neurological disorders [43,44,45,46]. The stability of circulating miRs can be attributed to their association with proteins such as AGO2 [47] and lipoproteins [48] or their containment in extracellular vesicles [49]. In this way, miRs are shielded from RNase enzymes and are stable in blood for up to 24 h [50]. Changes in circulating miR levels have been shown to be extremely sensitive to disease conditions and can outperform conventional biomarkers. For example, changes in circulating miR levels in response to disease states have been shown to be more rapid than those of mRNAs or proteins [46,51]. Oerlemans et al. demonstrated that changes in circulating miRs were detected earlier compared with that of troponin for the diagnosis and management of acute coronary syndrome [52].

HHT is diagnosed in combination by a clinical criteria known as the Curaçao criteria [53] and the molecular detection of known genetic mutations. Due to loci heterogeneity, technical challenges of molecular diagnostic techniques and de novo mutations, a clinical diagnosis is always required [53]. Additional biomarkers would greatly improve the diagnostic process as HHT is actually underdiagnosed [54]; they could also aid in the detection and management of clinical manifestations. The detection of AVMs is critical for the management of patient well-being. Approximately 50% of patients develop pulmonary AVMs (PAVMs), 80% develop hepatic AVMs (HAVMs), 10% develop cerebral AVMs (CAVMs) and 1% develop spinal AVMs (SAVMs) [2,55]. All AVMs are susceptible to rupture that can cause numerous life-threatening complications, including hemorrhagic and ischemic stroke, air embolism, congestive heart failure, cerebral abscess and seizure [55,56]. The timely detection of AVMs is critical to prevent serious and life-threatening complications, but current diagnostic screens are costly, relatively inaccessible and expose patients to unhealthy doses of radiation [57]. Circulating miRs could potentially provide a rapid, inexpensive, safe and relatively non-invasive screening test for the diagnosis of AVMs.

### 3.1. Elevated Circulating MiR-210 and PAVMs in HHT

A previous study from our laboratory identified a candidate circulating miR biomarker for the detection of PAVMs in HHT patients. Plasma miRs from HHT patients with PAVMs and healthy controls were profiled with a microarray analysis and a total of eight miRs were found to be dysregulated [58]. Select miRs identified by the array were validated with a reverse transcription quantitative polymerase chain reaction (RT-qPCR) and miR-210 was found to be significantly upregulated in plasma from HHT patients with PAVMs [58]. Additionally, miR-210 is a well characterized “hypoximir” that is robustly expressed in ECs under hypoxia [59,60]. The increased levels of circulating miR-210 identified in our study may be a result of PAVM-induced hypoxemia. It is also possible that this phenomenon may be a compensatory mechanism since EC overexpression of miR-210 has been shown to augment angiogenesis and tube formation [61]. Our study successfully identified a novel circulating miR biomarker that can potentially detect HHT patients only with PAVMs. However, it is necessary to improve the validity of this biomarker with a more clinically relevant sample size. Future work will aim to measure circulating miR-210 levels in HHT patients with treated PAVMs. Presumably, once an AVM is treated, typically by coil embolization, associated symptoms like hypoxemia should disappear. If miR-210 is in fact induced by hypoxia, then its levels should return to baseline once a PAVM has been treated. If this is the case, miR-210 stands to be an extremely sensitive and reliable biomarker for the detection of PAVMs in HHT.

### 3.2. Dysregulated Levels of Circulating MiR-205 and MiR-27a in HHT

Tabruyn et al. were the second to identify circulating miR dysregulation in HHT [62]. They conducted a miR microarray analysis on plasma from four HHT patients (two HHT1 and two HHT2) and identified 34 dysregulated miRs; 32 were upregulated, while two were downregulated. MiR-205 and miR-27a were selected for RT-qPCR validation in 24 HHT patients (11 HHT1 vs. 13 HHT2) and 16 controls. It was found that miR-205 was significantly decreased and miR-27a was significantly increased in HHT patient plasma. There were no significant differences between the expression of these miRs between HHT1 and HHT2 patients. This consistency highlights the potential of miRs as ideal candidates for the diagnosis of HHT.

**Figure 1:**
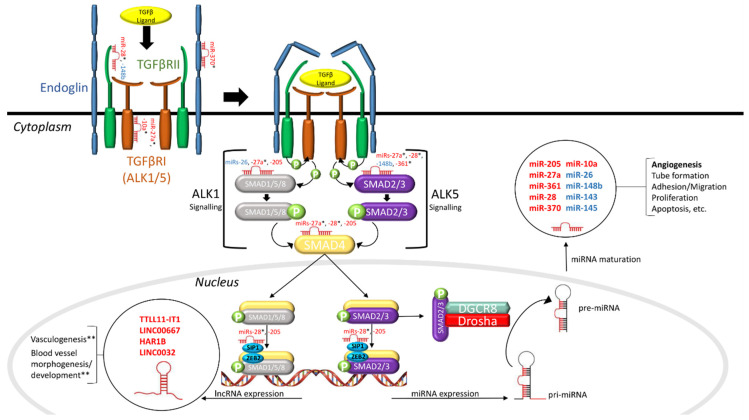
Interactions between non-coding RNAs and the TGFβ signaling pathway in hereditary hemorrhagic telangiectasia (HHT). As shown in this schematic diagram, dysregulated microRNAs (miRs) (in red) identified in HHT patients directly target a number of TGFβ signaling molecules, including SMADs and TGFβRs, and dysregulated lncRNAs (in red) found in HHT patients have a purported role in regulating vasculogenesis and vessel morphogenesis and development. SMAD2/3 can alternatively incorporate with the microprocessor complex to regulate the processing of pri-miRNA to pre-miRNA. Aberrant TGFβ signaling has been found to result in the altered expression of various miRs (in blue) that are involved in angiogenesis. The interaction between non-coding RNAs and TGFβ signaling establishes a narrative for their involvement in HHT pathogenesis. TGFβ: transforming growth factor beta; TGFβRI/II: TGFβ receptor I/II; ALK1/5: activin receptor-like kinase 1/5; miRNA: microRNA; lncRNA: long non-coding RNA; SMAD1/2/3/4/5/8: mothers against decapentaplegic homolog 1/2/3/4/5/8; SIP1: Smad interacting protein 1; ZEB2: zinc finger e-box binding homeobox 2; DGCR8: DiGeorge syndrome critical region gene 8; pri-miRNA: primary miRNA; pre-miRNA: precursor miRNA; “P”: phosphoryl group (* putative targets, ** predicted by bioinformatics).

MiR-27a is relatively well characterized and has been implicated in EC function [62,63], angiogenesis [63,64] and cancer [65]. Interestingly, a recent study has shown that miR-27a is hypoxia inducible [66]. Therefore, it is possible that the observed increase may be a result of HHT-related hypoxemia. However, Tabruyn et al. did not describe the clinical characteristics of enrolled patients. It would be interesting to explore the role hypoxia may play with regard to increased levels of miR-27a. Additionally, miR-27a has many putative targets in the TGFβ pathway, including SMADs (1/4/5/2), TGFβRI, ZEB2 and SP1 [67,68] (Figure 1). Future research should experimentally validate these targets in ECs. The involvement of miR-205 in angiogenesis and EC function is not well characterized; one study demonstrated that miR-205 regulates the expression of integrin β4, a major component of EC gap junctions [69]. Additionally, miR-205 has been shown to be involved in TGFβ signaling where it targets downstream transcription factors ZEB2 and SIP1 [70]. MiR-205 was shown to negatively respond to TGFβ1 stimulation in epithelial cells [71]. Tabruyn et al. further characterized the role of miR-205 in human umbilical vein endothelial cells (HUVECs). Overexpression of miR-205 in HUVECs decreased proliferation, migration and tube formation, while inhibition resulted in the opposite. Additionally, they described a role of miR-205 as a regulator of TGFβ signaling by targeting SMAD4 and SMAD1. They also demonstrated that miR-205 overexpression led to a significant increase in PAI-1 and a significant reduction in ID-1 mRNA levels. TGFβ signaling can be transduced by ALK1 and ALK5 pathways, where the former leads to EC activation, and the latter, EC quiescence. The effects of ALK1 and ALK5 signaling can predominately be attributed to the expression of PAI-1 and ID-1, respectively. They postulated that the observed reduction of circulating miR-205 levels is a result of reduced ALK1 signaling. They further suggested that the observed increase in circulating miR-27a is due to an increase in ALK5 signaling. This is based on the notion that an increase in ALK5 signaling is due to the reduction of ALK1 signaling in HHT. However, it has been demonstrated that ALK1 and ALK5 signaling maintain a very fine balance; if one pathway was reduced, the other would be equally reduced [9]. This was demonstrated in ECs cultured from HHT patient blood, known as blood outgrowth endothelial cells (BOECs) [72], and in ENG heterozygous mouse embryonic stem cells [73]. Thus, it is possible that the observed decrease and elevation in circulating miR-205 and miR-27a, respectively, may be the result of an alternate mechanism.

### 3.3. Dysregulated Circulating MiR-370 and MiR-10a in HHT1 and HHT2

Recently, Ruiz-Llorente et al. further identified dysregulated circulating miRs in HHT patient plasma [74]. In this study, the authors employed a miR-target prediction algorithm to specifically identify miRs that target ENG and/or ALK1. From the analysis, miR-370 and miR-10a were highly predicted to target ENG and ALK1, respectively, and miR-214 was highly predicted to target both ENG and ALK1. These miRs were selected for RT-qPCR validation in 34 HHT patients (17 HHT1 vs. 17 HHT2) and 16 controls. It was found that miR-370 was significantly decreased only in HHT1 patients compared with HHT2 patients and controls, while miR-10a was significantly increased only in HHT2 patients compared with HHT1 patients and controls. MiR-214 was not found to be significantly dysregulated.

MiR-370 has been experimentally validated to target ENG in ovarian cancer cells [75], yet its role in EC function and angiogenesis is unclear, as multiple reports have demonstrated conflicting evidence. Overexpression of miR-370 inhibited proliferation, migration and tube formation in human dermal microvascular ECs, retinal capillary ECs and HUVECs [76,77]. In contrast, miR-370 overexpression was shown to promote HUVEC proliferation, migration and tube formation to facilitate healing after finger amputation [78]. Additionally, miR-370 suppression by circular RNA circ_0003204 inhibited the proliferation, migration and tube formation of human aortic ECs [79]. It is unclear why this discrepancy exists, but suggests a far more complex role of miR-370 in EC function. MiR-10a has been shown to have anti-angiogenic effects in mouse umbilical vein ECs by targeting β-catenin [80]. It has also been shown to regulate VCAM1 expression in ECs under hemodynamic force by targeting GATA6 [81]. Therefore, an exact functional role in HHT pathogenesis remains unclear, but their potential as diagnostic biomarkers in HHT shows great promise. The ability of these miRs to distinguish between HHT1 or HHT2 patients could greatly improve the diagnostic process, as a number of HHT patients are asymptomatic or lack a known genetic mutation. Further research is required to validate these miRs in a more clinically relevant sample size and to directly confirm that these miRs indeed target ENG and ALK1 in ECs.

## 4. MiRs as Pathogenic Factors in HHT

MiRs have been demonstrated to play a pathogenic role in a variety of human diseases, including cancer, cardiovascular disease, autoimmune diseases and inflammatory diseases [22,23]. MiR profiling analyses have identified a plethora of up- and downregulated miRs in numerous types of cancers [24]. It has been shown that overexpressed miRs can act as “oncomiRs” by targeting tumor suppressors or by promoting proliferation and inhibiting apoptosis [82]. Additionally, the downregulation of certain “tumor suppressor” miRs, such as let-7, can also contribute to cancer pathogenesis [83,84]. A growing class of miRs known as “angio-miRs”, predominately expressed in ECs and responsible for the regulation of angiogenic processes, have also been shown to contribute to cancer and cardiovascular disease [27]. Given that HHT is a disorder characterized by angiogenic and EC dysfunction, it would not be surprising if dysregulated “angio-miRs” played a role in disease pathogenesis. There are numerous studies that have identified the involvement of miRs in TGFβ signaling. They have demonstrated that various components of the TGFβ pathway are targeted by miRs and that the pathway itself regulates miR biogenesis [85].

### 4.1. Canonical TGFβ Signaling in HHT

The TGFβ signaling cascade is initiated with the TGFβ superfamily of ligands (TGFβ1/3, BMPs, activin) binding in complex to two cell surface receptors; Type I (RI), such as ALK1/5, and Type II (RII) [86] (Figure 1). Upon ligand binding, these receptors form a heteromeric complex, activating their serine/threonine kinase activity [86]. Endoglin is an auxiliary co-receptor that can interact with the RI–RII–ligand complex to enhance ligand affinity [87]. Subsequently, RII will trans-phosphorylate RI, which in turn will phosphorylate the family of receptor-regulated SMADs (R-SMAD 1/2/3/5/8) [88]. The TGFβ pathway can propagate the signal through two cascades: SMAD1/5/8 or SMAD2/3. ALK1 signals through SMAD1/5/8, leading to EC activation [9,13]. ALK5 propagates the signal through SMAD2/3, leading to EC quiescence [9,13]. It has been shown that endoglin enhances the SMAD1/5/8 pathway and inhibits the SMAD2/3 pathway. Cascade selection is determined by the combination of ligand and receptors. Regardless of the selected cascade, both groups of R-SMADs interact with a common partner SMAD (Co-SMAD) known as SMAD4 [88]. This complex of R- and Co-SMADs translocates to the nucleus where it regulates the transcription of target genes [88].

### 4.2. Drosha and HHT Pathogenesis

Drosha is part of a protein complex called the microprocessor that is responsible for the cleavage of pri-miR into pre-miR [89]. The silencing of Drosha in HeLa cells results in the aggregation of pri-miRs and a decrease in pre-miRs [90]. Drosha is fundamental in the biogenesis of almost all miRs, but has also been shown to be involved in the regulation of mRNAs [90]. A few studies have demonstrated that Drosha can bind and cleave mRNAs, and can also associate with promoter regions to regulate specific genes [91,92,93]. In the TGFβ pathway, SMAD2/3 has been shown to incorporate with the microprocessor complex to regulate target miR genes [82,91] (Figure 1).

Kuehbacher et al. demonstrated that the knockdown of Drosha in HUVECs had a minimal effect on EC migration, viability and tube formation [94]. In vivo analysis of Drosha knockdown in a matrigel plug did not reveal significant effects on sprouting angiogenesis [94]. Even though Drosha knockdown had an insignificant effect on angiogenesis and EC function it reduced the expression of 29 miRs by 30% [94]. It is possible that the knockdown of Drosha activated compensatory mechanisms that mitigated its effects on EC function and angiogenesis. An inducible EC-specific knockout mouse model of Drosha would certainly provide a more robust analysis.

A study by Jiang et al. identified a higher prevalence of Drosha mutations in an HHT population and generated robust Drosha knockout animal models. Exome sequencing was performed on a total of 98 individuals; 23 confirmed HHT patients and 75 probands suspected to have HHT who lacked known pathogenic mutations [95]. Three heterozygous Drosha mutations (P32L, P100L and K226E) were detected in seven of the 98 individuals (~7%) compared with 0.04% in the general population [93]. An additional Drosha mutant (R279L) was found in a separate HHT family [93]. These Drosha mutants were analyzed in mouse embryonic fibroblasts (MEFs); interestingly, only the P100L and R279L mutants were shown to reduce expression of specific miRs, by approximately 17% and 35%, respectively, compared to wild type (WT) [95]. P100L and R279L were introduced into zebrafish embryos to investigate their angiogenic functions. Zebrafish with these two mutants developed vascular defects, including vascular permeability and a reduction of vascular density [95]. An EC-specific inducible knockout Drosha mouse model also demonstrated vascular permeability, disorganized vasculature and, interestingly, intestinal bleeding [95]. However, no AVMs were detected in either animal model [95].

The presence of Drosha mutations in HHT populations may contribute to disease pathogenesis and the observed clinical spectrum. BOECs derived from HHT patients with Drosha mutations may be a robust cellular model to profile and characterize affected miRs and mRNAs. Drosha itself has also been shown to regulate gene expression directly [90]. It would be interesting to identify Drosha-targeted genes and elucidate how they may contribute to HHT pathogenesis.

### 4.3. MiRs and HHT Pathogenesis

Few studies have characterized the role of miRs in HHT pathogenesis. However, numerous miRs have been identified to be regulated by and target components of the TGFβ pathway in a variety of tissues and disease conditions [85]. MiR-26 has recently been shown to be involved in vascular stability, where its knockdown in zebrafish led to vascular hemorrhage [96]. MiR-26 from ECs can regulate vascular smooth muscle cell (VSMC) differentiation in a paracrine manner [96]. It was shown that a decrease in miR-26 leads to an increase in its target, SMAD1, resulting in VSMC dysregulation and hemorrhage [96]. Another study demonstrated that the overexpression of miR-148b can improve migration, proliferation and angiogenesis in HUVECs by targeting SMAD2 and TGFβRII [97]. Subsequently, wound vascularization and healing were greatly augmented by a miR-148b mimic in a wound-healing mouse model [97]. MiR-148b inhibition greatly impaired wound healing, which was rescued by silencing SMAD2 [97]. A separate study demonstrated that VSMCs could regulate EC function via TGFβ-mediated secretion of miRs-143/145 [98]. Co-culture of VSMCs overexpressing miRs-143/145 with ECs greatly impaired EC function, while inhibition of the TGFβ pathway reversed this effect [98]. This study demonstrates how VSMCs can regulate EC activation/quiescence through the secretion of miRs in a TGFβ-dependent manner [98].

A previous study from our laboratory identified significantly decreased levels of miR-361-3p and miR-28-5p in peripheral blood mononuclear cells (PBMCs) derived from HHT patients [99]. PBMCs are mostly comprise lymphocytes and monocytes [100]. The pathogenic potential of PBMCs has been demonstrated in HHT1 mouse models. It has been shown that dysregulated TGFβ signaling adversely affects PBMC migratory capacity, which contributes to vascular dysplasia and prolonged inflammation [101,102]. The mRNA level of insulin-like growth factor 1 (IGF1), a putative target of miR-28-5p and miR-361-3p, was shown to be significantly upregulated in HHT patient-derived PBMCs. However, IGF1 plasma protein levels were not significantly different between patients and controls. It is possible that IGF1 could be overexpressed at angiogenic or inflammatory sites rather than systemically, contributing to the development of vascular dysplasia. IGF1 has been shown to play a significant role in augmenting angiogenesis in vitro and in vivo [103]. A previous study demonstrated that miR-361-3p is involved in the function of proangiogenic Tie2-expressing monocytes by targeting IGF1 [104]. MiR-28-5p has been shown to directly target IGF1 in hepatocellular carcinomas [105] and liver cancer stem cells [106]. Additionally, miR-28-5p and miR-361-3p have multiple putative targets in the TGFβ pathway, including SMADs, TGFβRII and SP1 [66,67] (Figure 1). It is possible that the observed decrease in miR-28-5p and miR-361-3p and increase in IGF1 may be a compensatory mechanism in response to reduced TGFβ signaling. Further research is required to understand the role these miRs play in PBMC dysfunction and ultimately HHT pathogenesis.

## 5. LncRNAs and HHT

LncRNAs are non-coding RNA species that are greater than 200 nucleotides in length and regulate gene expression post-transcriptionally [18]. LncRNAs can be divided into two functional groups based on their sub-cellular localization: (1) nuclear and (2) cytoplasmic. Nuclear lncRNAs can influence gene expression via various mechanisms, including chromatin remodeling, protein sequestration and the enhancement or dampening of promoter activity [107]. One of the ways that cytoplasmic lncRNAs can influence gene expression is by regulating the availability and stability of miRs by acting as miR “sponges” [107]. Although lncRNA mechanisms have been well characterized, evidence suggests that most lncRNAs are non-functional [107]. Nonetheless, a few lncRNAs have been implicated in vascular and endothelial cell biology. For example, the lncRNA MALAT1 was shown to promote EC proliferation and migration under hypoxic conditions [108]. Conversely, MEG3 was demonstrated to inhibit EC proliferation, survival and tube formation [108]. Singh et al. were the first to identify differential expression of lncRNAs in TGFβ1-stimulated HUVECs [109]. They demonstrated that 2051 and 2393 lncRNAs were significantly upregulated and downregulated, respectively. Of these, MALAT1 was upregulated the most (∼220-fold), contributing to its role in EC biology. Given the functional relevance of lncRNAs in EC biology, one would assume that they may play a role in HHT. Indeed, Tørring et al. sought to profile lncRNAs from nasal mucosa telangiectasias of HHT1 and HHT2 patients [110], and identified 42 lncRNAs that were significantly dysregulated (*p* < 0.001), including TTLL11-IT1, LINC00667, HAR1B and LINC0032, compared to non-telangiectasial nasal mucosa from the same HHT patients. However, none of the dysregulated lncRNAs have been characterized. Bioinformatic analysis revealed that these lncRNAs were enriched in HHT-related pathways, including vasculogenesis and blood vessel morphogenesis and development (Figure 1). Further research is required to characterize the role these dysregulated lncRNAs may play in HHT pathogenesis.

## 6. Conclusions and Future Research

The observation of ncRNA dysregulation in ECs and PBMCs from patients with HHT suggests that they may play a role in the pathogenesis of HHT. Further characterization of ncRNAs in other cell types involved in HHT, including VSMCs, pericytes and mononuclear cells is necessary to more fully understand the pathogenetic mechanisms controlling the disease and its progression. To date, the targets and functions of only a small fraction of ncRNAs identified in humans have been rigorously explored. With further research, ncRNAs may prove to have both diagnostic and therapeutic applications for those with HHT.
